# On the origin of non-specific binders isolated in the selection of phage display peptide libraries

**DOI:** 10.3389/fmicb.2025.1571679

**Published:** 2025-06-04

**Authors:** Babak Bakhshinejad, Andreas Kjaer

**Affiliations:** ^1^Cluster for Molecular Imaging, Department of Biomedical Sciences, University of Copenhagen, Copenhagen, Denmark; ^2^Department of Clinical Physiology and Nuclear Medicine, Copenhagen University Hospital-Rigshospitalet, Copenhagen, Denmark

**Keywords:** amplification, biopanning output, compositional bias, next-generation sequencing, non-specific binder, peptide library, phage display, target-unrelated enrichment

## Abstract

Over the recent decades, phage display has been used successfully to identify a variety of peptides with diagnostic and therapeutic applications. Despite the significant role of this technology in the pharmaceutical industry, the affinity selection of phage display peptide libraries through biopanning suffers from some limitations. The most significant drawback of phage display is the undesirable enrichment and isolation of phages whose displayed peptides have no binding affinity toward the target. Phages with high amplification rates constitute the most important category of non-specific binders. Amplification, which aims to increase the copy number of phages displaying target-specific peptides, acts like a double-edged blade and can also make a major contribution to the target-unrelated enrichment of non-specific binders, leading to compositional bias in the sequence content of the biopanning output. The cutting-edge breakthroughs fueled by the integration of next-generation sequencing (NGS) into phage display have led researchers to gain a deeper understanding of the information content of the phage population recovered from biopanning and how its peptide content changes during further rounds of selection and amplification. This body of vastly increasing information has shed more light on the complications encountered during library selection and opened new perspectives to obtain in-depth insights into amplification-associated bias in the selected phage display libraries, analyze biopanning data more rigorously, and devise more optimal protocols for phage display selections. This knowledge can finally provide a solid foundation for discovering promising target-specific binders in the evolutionary selection of phage display libraries.

## 1 Introduction

Combinatorial technology has provided a powerful means for generating libraries that contain an inexhaustible reservoir of ligands. The advent of this technology led to a paradigm shift in pharmaceutical research from identifying and characterizing single compounds to focusing on an expanded repertoire of compounds. In recent decades, advances in the field of molecular biology have resulted in the development of a variety of combinatorial biology techniques. Phage display is the most widely used approach in the toolbox of combinatorial biology methodologies. The first report describing this molecular technique was published in the mid-1980s by George Smith, at the University of Missouri (Smith, 1985). He cloned a fragment of the gene encoding the EcoRI restriction enzyme in *gene III* of the filamentous phage f1 and demonstrated that the insertion of enzyme fragments into the minor coat protein p3 (encoded by *gene III*) can be tolerated by the phage particle, with the expressed chimeric protein accessible on the phage surface for interaction with molecular partners. Further investigation indicated that the affinity capture with anti-EcoRI antibodies could result in the enrichment of recombinant virions from a mixture of insertless phage particles. The term “phage display” was later coined to describe the surface expression of foreign ligands on the surface of phages. Smith was co-awarded the Nobel Prize in chemistry in 2018 for this groundbreaking discovery.

## 2 Phage display peptide libraries: a treasure trove of peptides with clinical applications

Since the birth of phage display, this methodology has been successfully utilized to present ligands such as peptides, antibodies, and non-antibody proteins on the surface of phages. A pivotal feature of phage display is the physical link between phenotype (the surface-expressed molecule) and genotype (the nucleic acid sequence encoding the displayed molecule; Tikunova and Morozova, 2009). This means that while the ligand of interest is displayed on the phage surface, its encoding DNA is encapsulated within the same phage particle. This genotype-phenotype link allows for determining the amino acid sequence of any particular displayed ligand by directly sequencing its DNA insert in the phage genome. This feature also makes it possible to construct large and highly diverse libraries that can be screened for affinity toward a target by mimicking the natural evolution. The construction of peptide libraries has been a milestone in the history of phage display (Scott and Smith, 1990). These libraries contain up to millions of unique peptides presented on the surface of phage particles that can undergo evolutionary selection to discover peptides binding to a wide variety of targets.

Over the recent decades, peptide phage display has found versatile applications in a diverse range of biomedical areas such as drug discovery (Hamzeh-Mivehroud et al., 2013), drug delivery (Loi et al., 2013), tissue engineering (Bakhshinejad and Sadeghizadeh, 2014), molecular imaging (He et al., 2011), vaccine development (Aghebati-Maleki et al., 2016), and biosensor design (Xiaokang and Kun-Lin, 2013), among others. These applications have turned phage display into a powerful platform for identifying peptides with diagnostic and therapeutic potential. Consistent with this, there are a variety of reports demonstrating the successful application of peptides derived from phage display at the preclinical stage in different animal models (Konkalmatt et al., 2013; Jiang et al., 2015; Zhou et al., 2015; Fukuta et al., 2017; Qin et al., 2017; Kwak et al., 2020; Yamaguchi et al., 2020; El Fakiri et al., 2024). More importantly, peptide phage display has led to the development of several clinically approved drugs, such as romiplostim used for the treatment of chronic immune (idiopathic) thrombocytopenic purpura (ITP) (Molineux and Newland, 2010) and ecallantide used for the treatment of hereditary angioedema as well as the prevention of blood loss in cardiothoracic surgery (Lehmann, 2008). These achievements have conferred an outstanding position on phage display as an influential technology in the pharmaceutical industry.

## 3 Biopanning: mining combinatorial phage display libraries through affinity selection

The identification of peptides binding to a target of interest is performed through iterative cycles of library selection and amplification, named biopanning. A variety of targets, including but not limited to proteins (Zhang et al., 2015), carbohydrates (Loers et al., 2019), and nucleic acids (Cheng et al., 1996) can be used for this purpose. The targets employed in biopanning might have different modes of presentation. The target can be a molecule such as a recombinant protein immobilized directly (Li et al., 2019) or indirectly (through biotin-streptavidin interaction) (Sakamoto et al., 2017) on a solid support. Cultured cells can be used as the target in “whole-cell biopanning” (Bakhshinejad and Sadeghizadeh, 2022). Selection can also be performed on tissue extracts from animals or patients, known as “*ex vivo* biopanning” (Koivistoinen et al., 2013). Furthermore, *in vivo* biopanning has been carried out by injecting the peptide library into an animal or human subject (Arap et al., 2002; Li et al., 2012).

In biopanning, the target is incubated with the library to allow interaction between the target molecules and the virion members of the library. Some phages bind to the target. Then, the mixture of target-library is harshly washed to remove unbound or weakly bound phages. The bound phages are retrieved by elution that can be non-specific (e.g., pH change) or specific (e.g., competing ligand). The pool of eluted phages is amplified by infecting the host bacterium, typically an *Escherichia coli* strain. This amplified phage pool is used as the input library for the next round of affinity selection (Aghebati-Maleki et al., 2016; Smith, 2019). The selection and amplification steps of biopanning are carried out for multiple rounds to progressively enrich the population of phages displaying ligands with high binding affinity to the target and ultimately increase the proportion of target-specific phages over non-specific binders in the selection output. At the end of biopanning, a part of the genomic DNA of isolated phage clones that encodes the displayed peptide is subjected to sequencing to reveal the identity of peptides displayed by the phages (Aghebati-Maleki et al., 2016). Afterward, a variety of biochemical and biophysical methods, such as enzyme-linked immunosorbent assay (ELISA), surface plasmon resonance (SPR), immunofluorescence microscopy, and flow cytometry, are used to verify the binding capacity of identified peptides for the target *in vitro*. In addition, the target binding of peptides can be validated *in vivo* by testing them in animal models.

## 4 Next-generation sequencing and phage display: expanding horizons in peptide hunting

**Figure 1:**
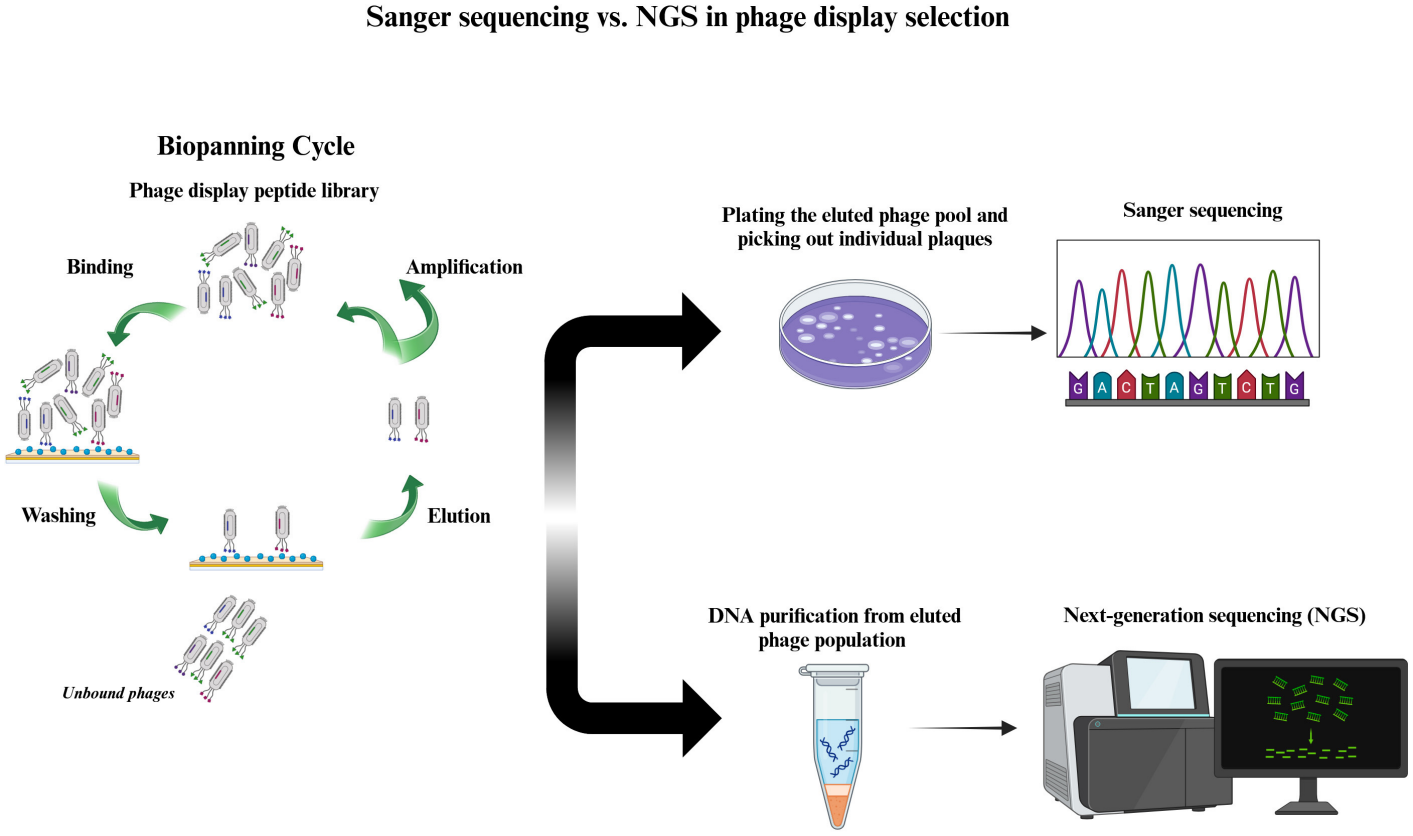
Phage display selection based on low- and high-throughput sequencing. After affinity selection of a phage display peptide library on the target through biopanning **(left)**, the eluted phage population is subjected to either low-throughput (right, above) or high-throughput (right, below) sequencing. In low-throughput sequencing, the phage pool recovered from biopanning is plated, and a limited number (usually tens) of plaques are randomly chosen. The DNA of these plaques is purified and subjected to Sanger sequencing. Another approach is to carry out PCR using viral plaques as the template, known as plaque PCR, and then subject the purified PCR product to Sanger sequencing. In contrast, in high-throughput sequencing, DNA is purified from a huge number of phages in the eluted phage population, PCR amplified, and subjected to NGS. In the NGS-based phage display, the DNA from thousands to millions of phage particles is sequenced, depending on the NGS platform's depth (represented by the number of reads) used for sequencing.

The sequencing strategy used to analyze the recovered phages from biopanning plays an important role in evaluating the results of a phage display selection. The traditional phage display selection is based on plating the phage suspension obtained from the elution step, randomly picking out a few viral plaques, and subjecting these plaques to the low-throughput sequencing (LTS) of the Sanger method. This conventional modality has been used for decades and is still widely used in phage display experiments. However, it presents a great disadvantage in that it covers a very small fraction of the whole sequence space of the selection output. Typically, tens, or at maximum hundreds, of clones are picked out and sequenced in a Sanger-based phage display. In recent years, a high-throughput sequencing (HTS) strategy called next-generation sequencing (NGS) has been integrated into phage display selection (Dias-Neto et al., 2009), increasing the efficiency and accuracy of binder discovery. Compared to Sanger sequencing, NGS covers a larger sequence space of the recovered phages from selection, enabling parallel sequencing of thousands to millions of clones (depending on the NGS platform that is used for the analysis of the results of selection) (Sloth et al., 2023). Figure 1 illustrates a comparison between Sanger- and NGS-based phage display selections. Although NGS cannot yet paint a complete picture of the selection output and its use is associated with some biases (generated during sampling and preparation of the genetic material of phages for sequencing), it allows for the inclusion of an incredibly larger number of phages eluted at the end of the selection and provides a solid foundation for more rigorous evaluation of biopanning data. In this manner, we can gain a more genuine understanding of the enrichment status of isolated hits, leading us to the successful discovery of promising target-binding peptide sequences in the selection of phage display libraries.

## 5 Isolation of non-specific binders: the greatest bottleneck of peptide discovery in phage display selection

Despite the crucial role of phage display in developing peptide diagnostics and therapeutics, this technology suffers from some limitations that have not received enough attention in the literature. Technically speaking, the greatest drawback of phage display is the enrichment of phages displaying target-unrelated peptides during selection, resulting in the undesirable isolation of non-specific binders at the end of biopanning. In this condition, instead of phages whose displayed peptides bind to the target, phages are isolated that display peptides with no binding affinity toward the target (Bakhshinejad et al., 2016). These non-specifically enriched target-unrelated binders fall into several categories:

1) Phages that bind to the selection background, and their displayed peptides do not bind to the target.
2) Phages that bind to the target, but their displayed peptides do not bind to the target.

The binding of both categories 1 and 2 to the target occurs through interaction between the phage body (a part of the phage surface other than the displayed peptide) on the one hand and either the background or the target molecule on the other hand.

3) Phages whose displayed peptides bind to the selection background, not the target.

The binding of category 3 occurs through interaction between the displayed peptide on the phage surface (without involvement of the phage body) and the background.

**Figure 2:**
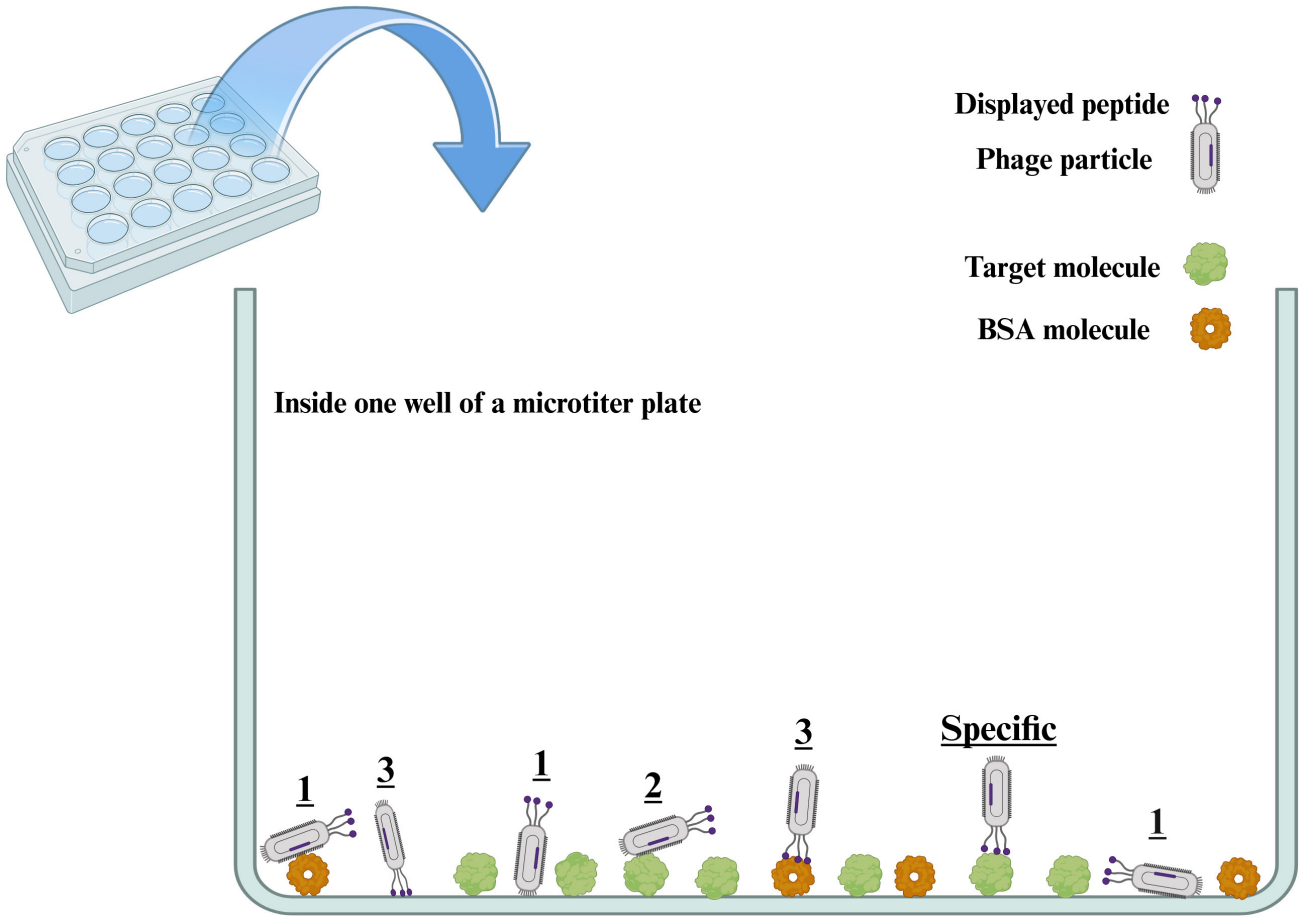
Possible interactions between the phage particles of the library and the target molecule immobilized on the surface of the well of a microtiter plate. Three categories of non-specific molecular interactions, denoted by numbers, are likely to happen in the well of a microtiter plate between the phage particles and either the background or the target molecule (e.g., a recombinant protein). Category 1 refers to the non-specific interactions between the phage particle and the background (the microtiter well surface and BSA). Category 2 refers to the non-specific interactions between the phage particle and the target molecule. In both interactions of categories 1 and 2, parts of the phage surface other than the displayed peptide are involved in interaction with either the background or the target. In addition, phage particles might have a variety of orientations for these non-specific interactions (two different orientations are shown for the interaction of category 1 phages with the well surface). Category 3 refers to the non-specific interactions between the displayed peptide and the background (the well surface and BSA). The category indicated as “specific” represents the interactions between the phage surface-displayed peptide and the target molecule. Out of all these possible interactions, only the “specific” type is desired and leads to a successful phage display selection. Depending on the orientation of interacting molecules toward each other, one single phage particle might be concurrently involved in more than one type of interaction. To have a more understandable visualization of interactions, we avoided showing all the components of the selection system, and only the surface of the solid support (microtiter well) and BSA are illustrated as the most important components of the background. Also, the dimensions of phage particles, target molecules, and BSA molecules have been depicted similarly to each other. However, in reality, phage particles are macromolecular complexes that have significantly larger dimensions than target and BSA molecules. BSA: bovine serum albumin.

In these non-specific interactions, background means all of the components of the selection system excluding the target, which include the surface of the solid support (microtiter plates, tubes, Petri dishes, magnetic beads, flasks, and column matrices), biotin and streptavidin (in the case of having biotinylated targets immobilized on streptavidin-coated solid phase), bovine serum albumin (BSA) or skimmed milk used as the blocking buffer to fill the empty surfaces of the solid phase, metal ions utilized to immobilize the His-tagged protein targets, protein A and G used to capture antibody targets, and so on (Menendez and Scott, 2005; Bakhshinejad et al., 2016). Figure 2 schematically illustrates the possible non-specific interactions that might happen in the well of a microtiter plate (as the most widely used solid support for biopanning), leading to the emergence of categories 1, 2, and 3 of non-specific binders described earlier. The background might have a broader definition in whole cell, *ex vivo*, and *in vivo* biopanning schemes. In these cases, off-target molecules on the surface of target cells, as well as off-target cells in tissue extracts and the body of animal or human subjects, can also be considered as background. The displayed peptide involves a small area on the whole phage surface. Given the broader dimensions of the whole phage body vs. the displayed peptide, as well as the larger abundance of the molecules of background elements vs. the target, category 1 is the most important non-specific interaction and is much more likely to take place compared to the other non-specific interactions, in particular category 3, and even compared to the specific interaction between the displayed peptide and the target. As a result, the non-specific interaction of category 1 is one of the major reasons for the failure of phage display selections.

A vast number of peptides have been reported as binders to the elements of the selection apparatus. A comprehensive list of these peptides can be found in (Menendez and Scott, 2005; Vodnik et al., 2011; Bakhshinejad et al., 2016). One of the most informative clues leading to the discovery of these peptides as binders to the constituents of the selection system, not the target of interest, has been their repeated isolation in library selections on different targets, sometimes with completely unrelated structural and functional identities. An in-depth analysis of the sequence features of these selection-related non-specific binders has resulted in identifying a number of consensus binding motifs. Table 1 contains a summarized list of the most important consensus sequence motifs indicated to bind to the components of the selection system in phage display studies. In some cases, further investigation of these peptides has revealed a correlation between biochemical signatures and binding to the component of interest in the selection system, elucidating the mechanistic details of unintended interactions that occur during biopanning between these target-unrelated peptides and their off-target binding partners. Polystyrene is the most common material used as the solid support in phage display experiments, as well as in some of the downstream assays for binding characterization of isolated peptides. Many peptides with polystyrene-binding capacity have been identified in phage display studies. It has been demonstrated that hydrophobic amino acids, in particular aromatic residues such as phenylalanine, tyrosine, and tryptophan, play major roles in interaction with polystyrene (Adey et al., 1995; Menendez and Scott, 2005). In this regard, we identified a heptapeptide sequence containing four hydrophobic residues as a specific binder to the polystyrene surface of the microtiter plate. This peptide was originally isolated in biopanning on colon cancer cells. However, a rigorous assessment of its binding properties through a variety of bioinformatic and experimental approaches confirmed its specific binding to polystyrene (Bakhshinejad and Sadeghizadeh, 2016). In terms of structural features, simulating the secondary structure of a polystyrene-binding peptide by molecular modeling has shown that stable structures of α-helix and β-turn contribute to peptide binding to polystyrene. The side chains of aromatic residues in these structures can create strong hydrophobic interactions with the benzene ring of polystyrene (Qiang et al., 2017).

**Table 1:** A list of consensus sequence motifs identified as binders to the different components of the selection apparatus in phage display studies.

**Binding element in the selection system**	**Consensus sequence motif**	**Reference(s)**
Plastic	WXXW	Adey et al., 1995; Gebhardt et al., 1996
Plastic	WHXW	Caparon et al., 1996
Plastic	WXXWXXXW	Gebhardt et al., 1996
Plastic	FHXXW	Anni et al., 2001
Streptavidin	HPQ	Devlin et al., 1990; Giebel et al., 1995
Streptavidin	GD(F/W)XF	Roberts et al., 1993
Streptavidin	PWXWL	Roberts et al., 1993
Streptavidin	EPDW(F/Y)	Caparon et al., 1996
Biotin	WXPPF(K/R)	Saggio and Laufer, 1993
Albumin	DICLPRWGCLW	Dennis et al., 2002
Divalent metal ions	HTHHHT HHHHPT HHHHHT HHHHTN HHTHSL	Mathonet et al., 2006
Protein A	WT(I/L)XXHR	Jesaitis et al., 1999
Fc region of IgG	SS(I/L)	Krook et al., 1998; Ehrlich and Bailon, 1998; Menendez and Scott, 2005
Fc region of IgG	GELVW	Delano et al., 2000
Fc region of IgG	WI(S/P)(S/Q)XDW	Glee et al., 1999
Bovine IgG	QSYP	Jacobs et al., 2003; Bresson et al., 2003; Menendez and Scott, 2005

The unintended isolation of selection-related non-specific binders can reduce the success rate of phage display selection in identifying target-specific ligands. Thus, it is highly critical to minimize the negative contribution of these non-specific interactions to the library selection. Enhancing the stringency of blocking and washing steps during successive rounds of biopanning is a frequently utilized strategy to reduce non-specific binding of the library virions (Nafian et al., 2019; Bakhshinejad and Sadeghizadeh, 2022). More importantly, pairing positive selection (biopanning on the target of interest) with negative selection is widely used to lessen the likelihood of the isolation of selection-related non-specific binders. In negative selection, the input phage library is pre-incubated with a well containing all components of the selection system except the target. The primary goal of this approach, which is also known as subtractive selection, is to deplete the library of as many non-specific binders as possible. Library subtraction is very common in whole-cell biopanning, in which one or more types of control cells are included in the negative selection step of biopanning (Cieslewicz et al., 2013; Bakhshinejad and Nasiri, 2018). However, it is worth mentioning that the extreme stringency of washing and blocking, as well as the over-stringent subtraction of the library, can result in a compromised diversity of the library, eliminating a significant number of phages that display potential target-binding peptides. Therefore, highly stringent selection steps and too stringent library subtraction should be avoided to preserve the functional diversity of the library.

4) Phages that have high propagation rates. The isolation of these phages at the end of biopanning is mainly due to their propagation advantage over other clones of the library, not because of the binding affinity of their displayed peptides to the target.

The high amplification rate of phage clones can be either sequence-dependent (intrinsic to the displayed peptide) or sequence-independent (extrinsic to the displayed peptide; Zade et al., 2017). The former originates from peptides or motifs that increase the amplification rate of phage clones (Sinkjaer et al., 2025). The latter results from alterations in the genome that enhance the amplification rate of phage clones. Three types of genomic changes, including point mutations (in the regulatory regions of the phage genome; Nguyen et al., 2014), genomic deletion (Kamstrup Sell et al., 2022), and genomic rearrangement (Thomas et al., 2010), have been indicated to contribute to the higher amplification rate of phage clones. The distinction between selection-related (categories 1, 2, and 3) and propagation-related (category 4) non-specific binders should not be interpreted as meaning that a selection-related non-specific binder cannot have a high amplification rate. A phage clone with a propagation advantage, and consequently a higher copy number, is very likely to get a higher chance for interaction with the different components of the selection background through either the phage body or its displayed peptide, leading to the target-independent enrichment of these phages during biopanning. In section 7, sequence features in displayed peptides that contribute to the amplification rate of phage clones, and in section 8, some approaches that can be used to minimize the undesirable isolation of propagation-related non-specific binders will be discussed.

## 6 Amplification and bias in the sequence composition of phage display libraries: a double-edged blade in binder identification

It is well established that compositional bias is an inherent characteristic of phage display libraries. The earliest reports on bias in phage libraries were published in the first years after the introduction of phage display technology (Peters et al., 1994; Malik et al., 1996). Compositional bias is defined as the non-random representation of amino acids and peptides in the naïve library before the phage pool is subjected to any selective pressure to enrich target-binding sequences. Divergence from randomness is shown by the under- and overrepresentation of amino acids at different positions of the displayed peptides as well as the non-uniform distribution of peptide frequencies in the whole library that brings about the under- and overrepresentation of peptides characterized by a wide variation in their copy numbers in the naïve library (Sloth et al., 2022). Observing bias in the naïve phage display libraries, which have not gone under selection yet, reinforces the notion that some part of the bias originates from the phage life cycle and its interaction with the host bacterium. However, it has been demonstrated that amplification can also contribute to increasing the extent of bias in the sequence composition of phage display peptide libraries (Matochko et al., 2014). Amplification is conducted at the end of each selection round and is considered an essential step of any phage display selection experiment. The amplification-induced bias is caused by the differences in the amplification rates of phage clones existing in a library. The target-unrelated enrichment of fast-propagating clones taking place during amplification is a hallmark characteristic of compositional bias in phage display libraries.

Amplification aims to increase the copy number of phages remaining at the end of selection. The fundamental presumption in phage display is that most, or at least a large number of, phages in the selection output are viruses that display ligands binding with a spectrum of affinities (low, medium, and high) to the target, and thus, amplification largely multiplies phage clones displaying target-specific binders. However, recent findings in the field of phage display have cast doubts over this long-lasting standpoint, suggesting the idea that a small fraction of the selection output is populated by phages that display target-specific binders. These novel insights on the sequence composition of selection output have been shaped by using high-throughput sequencing technologies to analyze the phage pool recovered from library selection. In a report, we indicated that even by applying a highly stringent selection of a 7-mer library on a protein target (recombinant human CD4), thousands of unique peptides (around 20,000) can still be found in the NGS dataset obtained from the analysis of the third round of biopanning (Sell et al., 2024). The presence of a high abundance of sequences in the biopanning output has also been demonstrated in other studies using NGS for the analysis of biopanning data (Brinton et al., 2016; Vekris et al., 2019; Asar et al., 2020; Mishra et al., 2021; Miki et al., 2022; Sevenich et al., 2022; Jirwankar and Dighe, 2023). It is obvious that there are a limited number of binding sites on the target molecule that can interact with a few different peptides from the library. Therefore, only a small number of peptides in the recovered phage population can bind to the target, and a large number of peptides isolated in biopanning are very unlikely to have specific interactions with the target molecule. These findings highlight that a large fraction of the biopanning output is still composed of non-specific binders, leading us to the conclusion that although amplification is intended to enlarge the target-specific fraction of the selection output, it concurrently makes a major contribution to the enrichment of the target-unrelated portion of the selection output. Another drawback of amplification is the overwhelming increase in the insertless, also known as wild-type (WT), clones of the phage pool (Sinkjaer et al., 2025). These clones are an artifact of library construction and do not display any peptide. The M13 phage is the most commonly used bacteriophage for the display of different molecules, including peptide ligands. Peptides are often displayed as fusions to the p3 of M13 phage. This protein is involved in the infection of the host bacterium by the phage (Click and Webster, 1997). Inserting a displayed peptide into p3 is disadvantageous for the assembly and infection of M13 phage particles. In contrast, insertless phage clones have a selective advantage for amplification over peptide-displaying phages, leading to their exponentially increased copy number during amplification between rounds of selection (Smith, 1985). Consistent with these findings, we can deduce that amplification has a dual role in the evolutionary selection of phage display libraries and acts like a double-edged blade, playing roles in the enrichment of both categories of target-specific and non-specific binders.

To quantitatively address the consequences of amplification-induced bias and minimize its negative impact on biopanning results, peptide sequences that are displayed on fast-propagating phage clones and thus non-specifically enriched during phage display selection should be identified and removed from the list of peptides synthesized for downstream binding characterization assays. This can be achieved by amplifying the naïve library with no selection (on the target of interest) between amplifications and subjecting the amplified phage pool(s) to NGS analysis. Target-independent serial amplification of the naïve library should be conducted for the same number of rounds as biopanning. Despite being highly useful, few studies have reported this strategy as an efficient means to identify sequences whose target-independent enrichment in the biopanning output arises from the higher amplification rate of the respective phage clones, not specific binding to the target (Liu et al., 2015; Miki et al., 2022). A more meticulous approach to quantitatively characterize the magnitude of amplification-associated bias and reduce its impact on deviating the interpretation of biopanning data is to calculate the enrichment factor (EF) for each sequence gained from NGS analysis. At first, the relative frequency of a sequence is quantified by dividing the count of each sequence by the total counts in each sample (biopanned and amplified libraries), and then the sequence EF is calculated by the ratio of its relative frequency in biopanning output vs. amplification output (Miki et al., 2022). Calculating the EF of sequences during successive rounds of target-dependent biopanning and target-independent serial amplification can help in tracking the evolution of peptide sequences under selective pressures for both target binding and amplification capacity. Monitoring alterations in the EF profile of a large number of sequences in NGS datasets can provide invaluable insights into the selection of genuine target-specific binders.

As noted at the beginning of this section, bias is inherently present in the peptide content of naïve phage display libraries before undergoing any selection and amplification. The existence of this initial bias can have a cascading effect, leading to increased skewness in the peptide composition of phage pools during the subsequent selection and amplification steps of biopanning. One powerful strategy to mitigate this inherent bias and restrict its negative impact on the unintended selection of non-specific binders is to optimize the different steps of library construction as well as characterize the constructed library before biopanning by utilizing NGS. Optimization of the library construction steps includes selecting appropriate codon schemes, refining the synthetic chemistry of the library oligonucleotides, and improving the efficiency of cloning and transformation. Rather than using the fully degenerate NNN codon, where each position can be any of the four nucleotides, randomization to construct phage display peptide libraries is typically performed using a reduced genetic code. NNN codon randomization suffers from a high frequency of stop codons (3 out of 64), leading to a diminished number of full-length functional clones and a severe codon redundancy (e.g., 6 codons for leucine, arginine, and serine), resulting in a major imbalance in the amino acid representation of the library. In contrast, randomization schemes like NNB (B: C, G, or T), NNK (K: G or T), and NNS (S: G or C) encode all twenty amino acids and only a single stop codon while reducing the total number of codons from 64 to 48 in NNB and 32 in NNK and NNS schemes, respectively (Sieber et al., 2015). Using randomization schemes with reduced genetic code provides the advantage of limiting the number of codons per amino acid, allowing for a more uniform distribution of amino acids into the peptide library, as well as decreasing the frequency of stop codons. As a result, libraries constructed with reduced genetic code yield superior sequence diversity compared to those based on the standard genetic code. Interestingly, analysis of the relative concentration of the four nucleotides at each position within the NNK scheme has revealed significant distortions in the efficiency of incorporation of nucleotides during the chemical synthesis of the library, represented by more efficient incorporation of G (guanine) compared to other nucleotides. This biased chemistry of oligonucleotide synthesis results in a skewed amino acid composition of the library, which is particularly relevant in the K position, causing an overrepresentation of amino acids encoded by codons with G in the third position. This could be rectified using compensating oligonucleotide mixtures calibrated by mass spectroscopy and utilizing the calibrated nucleotide ratios to construct phage display peptide libraries (Ryvkin et al., 2018). However, the degenerate nature of the genetic code in all of the mentioned schemes can impose limitations on the quality and diversity of the library. An effective approach to address this restriction and eliminate codon redundancy involves constructing libraries in which each amino acid is represented by exactly one codon. These libraries have 20 codons for 20 amino acids, also known as 20/20 libraries. A major advantage of 20/20 libraries is the complete avoidance of stop and non-sense codons (Yan et al., 2014), which has been indicated to enhance the functional diversity of phage display peptide libraries (Krumpe et al., 2007). The most common method for generating such libraries is the trimer approach, where oligonucleotides are synthesized by assembling prefabricated trinucleotide phosphoramidites (trimers), each corresponding to a specific amino acid (Baldwin et al., 2008; Suchsland et al., 2018). Alternatively, 20/20 libraries can be constructed by ProxiMAX randomization, which uses standard unmodified oligonucleotides and constructs the library with a different assembly strategy. In ProxiMAX, the combinatorial peptide library is built codon-by-codon via ligation (Ashraf et al., 2013; Frigotto et al., 2015). Another important correction that can reduce bias during library construction is to optimize the efficiency of cloning and transformation. The inefficient linearization of circular M13 vectors, which are recognized as the most widely used platform for the construction of phage display peptide libraries, can lead to a high frequency of insertless clones in the naïve library. This problem can be addressed by increasing the stringency of the digestion of vector molecules or employing purification techniques such as gradient density centrifugation to separate fully digested vector molecules (Brakmann and Schwienhorst, 2006; Sloth et al., 2022). Furthermore, the efficiency of transformation needs to be maximized to ensure that more unique clones are introduced into host bacteria and a broader sequence space is represented in the library. It is important to note that the highest experimentally attainable diversity of phage display libraries is limited to 10^9^, primarily because of constraints in the transformation efficiency of *Escherichia coli* cells. However, the actual diversity of the library that can be obtained may be even lower due to the presence of stop codons, frameshift mutations, and insertless clones. Therefore, the functional diversity of phage display peptide libraries is generally estimated to fall within the range of 10^8^-10^9^ (Sloth et al., 2023). The host bacterium used for library transformation is also important and needs to be a suppressor strain. The use of suppressor strains plays a crucial role in addressing issues associated with stop codons that are commonly observed when using degenerate codon schemes. Stop codons prematurely terminate translation, leading to the abortive termination of the phage fusion coat protein. A suppressor strain is a genetically modified bacterium that contains special tRNA genes capable of suppressing and reading through stop codons during translation by recognizing a stop codon and inserting an amino acid instead. In amber suppressor strains, glutamine is inserted when UAG is present in the mRNA transcribed from the displayed peptide-encoding DNA. By comparing the percentage of peptides containing UAG between type 88 phage display libraries produced in bacteria with or without suppression, it was demonstrated that using an amber suppressor strain can significantly reduce abortive termination of the recombinant fusion phage and decrease the frequency of phages containing UAG-bearing inserts. This ensures a more functional diversity of the library (Ryvkin et al., 2018). Quality control of the constructed library before biopanning is also significant to portray a more thorough picture of its compositional characteristics. The huge amount of data provided by NGS enables a comprehensive characterization of the peptide composition in the naïve library. This quantitative analysis can help in detecting some artifacts of library construction (e.g., the abundance of wild-type clones or stop codons) and offering insights into the overall quality of the library (Sloth et al., 2022). NGS datasets can be searched to identify overrepresented sequences, which are most likely to be propagation-related peptides, and discover peptides containing consensus sequence motifs that bind to the different components of the selection apparatus (such as those sequences shown in Table 1). The NGS-based discovery of such propagation-related and selection-related peptides in the naïve library allows for removing a significant number of potential non-specific binders from future biopanning data.

## 7 Sequence features of displayed peptides at amino acid, motif, and peptide levels: contributors to the amplification rate of phage clones

Accumulating knowledge in the field of phage display reinforces the notion that sequence features of a displayed peptide at the amino acid, motif, and peptide levels contribute to the amplification rate of the respective phage clone. Peptide sequences that are incompatible with infectivity, biosynthesis (nucleic acid replication and protein synthesis of phage by the replication, transcription, and translation machinery of the host cell), and assembly of phage particles (at the membranes of the host cell) are underrepresented, whereas peptide sequences that enhance fitness for the different steps of the phage life cycle provide a selective advantage for propagation and thus are overrepresented in the library (Zade et al., 2017). The sequence analysis of M13-based libraries, as the most widely used phage display peptide library for biopanning experiments, by using both low-throughput and high-throughput sequencing highlights that there is a correlation between sequence signatures in a displayed peptide and the propagation capacity of the corresponding phage clone. In this regard, overrepresentation of proline (except the first position), threonine, and histidine, as well as underrepresentation of cysteine, glycine, and positively charged residues, in particular arginine, have been reported in various studies as a general sequence pattern for naïve phage display peptide libraries (Rodi et al., 2002; Krumpe et al., 2006; Ac't Hoen et al., 2012; Sloth et al., 2022). These observations in the overall abundances of different amino acids are attributed to the functional impact of these sequence features on the different steps of phage morphogenesis, thus decreasing or increasing the amplification rate of phage clones. Proline has been suggested to contribute to the rigidity of the polypeptide backbone, thereby playing a crucial role in limiting the number of possible conformations that a short peptide can adopt and stabilizing its active fold (Kini and Evans, 1995). This structural function, which is significant for the activity of virus-displayed peptides, can explain the overrepresentation of proline in phage display peptide libraries. Interestingly, the N-terminus of the displayed peptide, in particular the first position, harbors the majority of altered amino acid abundances. The N-terminus of a phage-displayed peptide is in the vicinity of the cleavage site of signal peptidase, an enzyme that cleaves signal peptide from the peptide-pIII fusion and plays an important role in the proteolytic processing of phage coat proteins. This explains why proline is underrepresented at the first position, as the presence of proline at position 1 of the displayed peptide inhibits the cleavage activity of signal peptidase on a peptide-pIII fusion protein (Barkocy-Gallagher and Bassford, 1992; Nilsson and Von Heijne, 1992). Arginine is underrepresented at the N-terminus because its presence at this part of the displayed peptide interferes with the translocation of pIII across the inner membrane of the host bacterial cell, leading to the inhibited secretion of phage particles from the host bacterium (Yamane and Mizushima, 1988; Peters et al., 1994). The extreme underrepresentation (censorship) of cysteine originates from the tendency of unpaired cysteines to create intramolecular disulfide bonds with internal cysteine residues of pIII, potentially disrupting the infectivity or assembly of filamentous phage particles (Mcconnell et al., 1996). In addition to the above-mentioned findings at the amino acid level, there is also some information in the literature that underscores the contribution of a stretch of amino acids or motifs in the displayed peptide to the amplification rate of the relevant clone. In support of this notion, it has been found that a proline-rich peptide sequence with large hydrophobic residues fused to the pVIII of the M13 phage facilitates the assembly and secretion of phage particles, giving rise to a selective advantage for propagation with the resultant isolation of phages at the end of library selection. The selective advantage of phages for amplification is explained by the fact that peptides containing this sequence feature that are fused with pVIII can create a compact structure on the phage body and ideally fit into the groove between two contiguous pVIII molecules on the phage surface, leading to an increased secretion of the phage particle (Iannolo et al., 1997). The massive repertoire of peptide sequences generated by NGS enables searching for sequence motifs associated with the propagation advantage of phage clones. For this purpose, we carried out an in-depth motif discovery analysis on the naïve and amplified phage pools of two different lots of a phage display peptide library. A motif search in the naïve library was conducted to recognize background motifs, which can be falsely identified as amplification-related motifs, and filter them out of the final list of genuine motifs. Our extensive analysis of the large NGS datasets led to the identification of a number of motifs non-existent in the naïve library but emerged and conserved during successive rounds of amplification, providing evidence for the probable contribution of these motifs to the enhanced amplification rate of the respective phage clones (Sinkjaer et al., 2025).

There is scarce information in the literature about the role of the secondary structure of displayed peptides in the propagation capacity of phage clones. In this context, peptides that form β-turn and α-helix structures on pIII have been suggested to result in higher and lower amplification rates of phage clones, respectively (Rodi et al., 2002). This has been ascribed to the effect of three-dimensional conformation formed by these secondary structures on the proteolytic activity of signal peptidase and, consequently, the catalytic processing of phage coat protein. However, our detailed analysis of the secondary structure in the massive number of peptides obtained from NGS, which was performed by determining the number of peptides containing a variety of motifs already known to be associated with β-turn formation and then calculating the enrichment score of these motifs during rounds of amplification, highlighted the lack of such a correlation between the β-turn structure in the displayed peptide and the selective advantage of the relevant phage clone for propagation (Sinkjaer et al., 2025).

**Figure 3:**
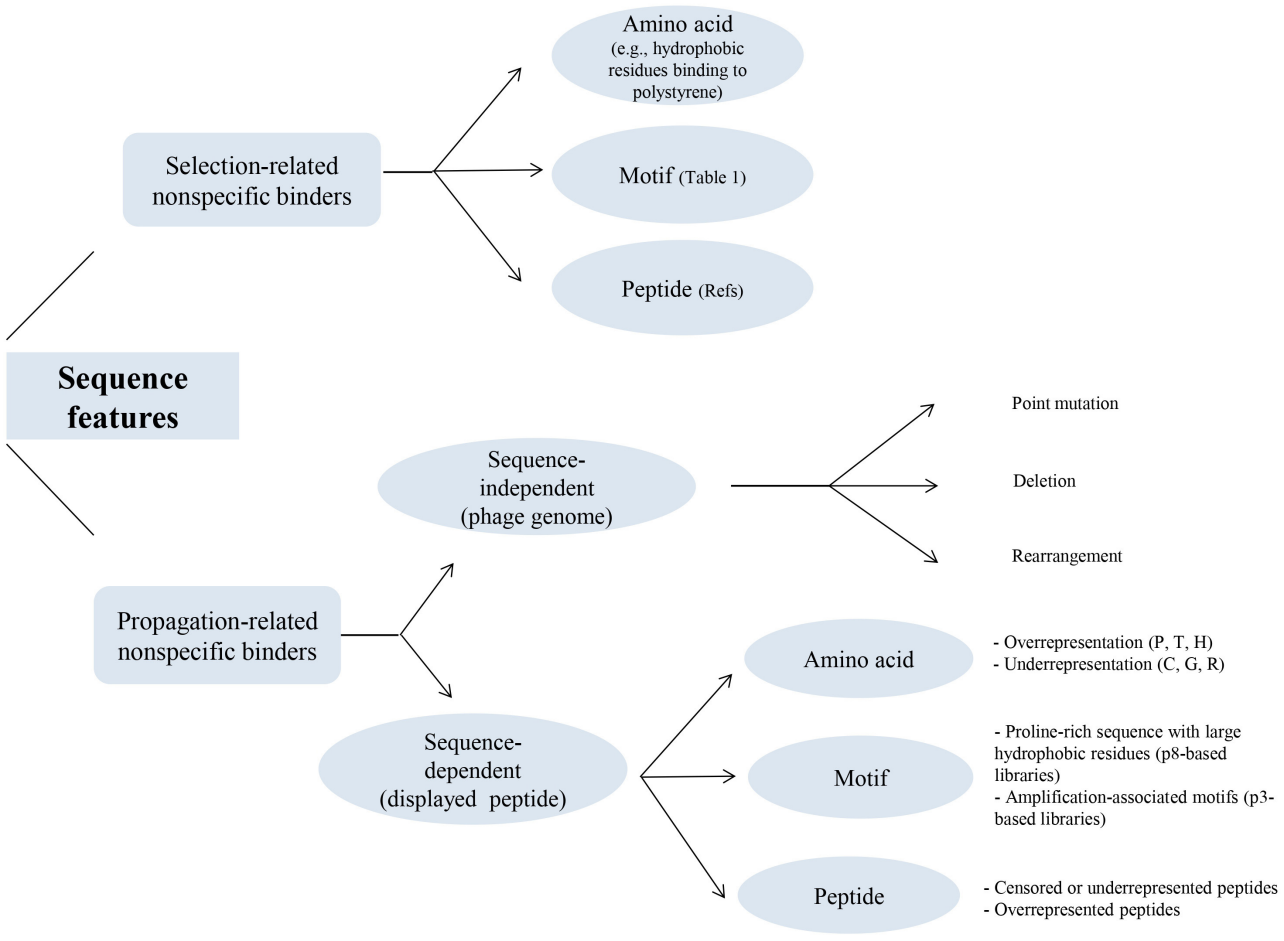
Sequence features identified in the non-specific binders isolated in phage display selection studies. The figure provides a classified summary of the sequence features that have been demonstrated to contribute to the unintended enrichment of both categories of selection-related and propagation-related non-specific binders during the affinity selection of phage display peptide libraries. For each category of these non-specific binders, sequence features are shown at amino acid, motif, and peptide levels. In selection-related non-specific binders, hydrophobic amino acids, in particular aromatic residues, have been indicated to be involved in interaction with the polystyrene surface of microtiter plates, which is the most common type of solid support material in phage display selections. In addition, a few consensus motifs (summarized in Table 1) as well as a large number of peptide sequences (of varying lengths and conformations) that can be found in these references (Menendez and Scott, 2005; Vodnik et al., 2011; Bakhshinejad et al., 2016) have been identified as binders to the different components of the selection apparatus. In propagation-related nonspecific binders, the target-unrelated enrichment of phage clones during biopanning has been known to be (1) extrinsic to the displayed peptide (sequence-independent) that originates from the different types of sequence alterations in the phage genome, including point mutation, deletion, and rearrangement, and (2) intrinsic to the displayed peptide (sequence-dependent) that originates from amino acids, motifs, and peptides whose absence or presence (represented by censorship/underrepresentation or overrepresentation) in the phage-displayed peptide leads to changes in the amplification rate of the respective phage clone. P, proline; T, threonine; H, histidine; C, cysteine; G, glycine; R, arginine.

Figure 3 illustrates a classification of the sequence features shown to be involved in the unintended enrichment of selection-related and propagation-related non-specific binders. This figure provides a summary of the sequence features at amino acid, motif, and peptide levels described in sections 5 and 7.

## 8 Reducing amplification rounds: en route to a minimized bias in the peptide content of the selection output

The growing body of evidence supporting the dual role of amplification in the evolution of the sequence composition of phage display libraries can lead to a transformation in our understanding of the conventional phage display selection, which is based on performing multiple rounds of selection and amplification, and raises the question of how the negative consequences of amplification can be minimized. It has been found that a significant enrichment can be observed after the first round of affinity selection (Ac't Hoen et al., 2012). In addition, we have recently indicated that additional rounds of amplification result in a remarkable enhancement in the percentage of insertless clones, with the largest increase observed in the third (as the last) round of amplification (Sinkjaer et al., 2025). Amplification also gives rise to a decline or collapse in the diversity of phage display libraries (Matochko et al., 2012). This can be ascribed to the evolutionary competition between slow- and fast-propagating phage clones. During amplification, fast-propagating phages acquire a higher copy number compared to slow-propagating ones. As already mentioned, these changes in the copy number derive from discrepancies in the amplification rates of clones but are not dependent on the binding capacity of their displayed peptides for the target. Due to lower copy numbers, slow-propagating phages are more likely to be outcompeted by clones with a propagation advantage and, thus, be eliminated from the phage pool, though they might display target-binding peptides (Bakhshinejad et al., 2016). Based on these lines of evidence, additional rounds of selection and amplification might not provide an added advantage for the identification of target-specific binders. Instead, performing parallel selections (duplicate, triplicate, or even quadruplicate, depending on the available resources) with only one round of amplification, comparing the sequences acquired from multiple selections, and finally choosing identical peptides between parallel selections might be considered a more rigorous approach for the identification of promising peptides. Furthermore, parallel selections can reduce variability that might happen in different biopannings in terms of the stringency of binding conditions. Avoiding further rounds of selection and amplification can also considerably shrink the fraction of the selection output populated by the insertless clones. In line with this, duplicate phage display selection (coupled with high-throughput sequencing and analysis of NGS datasets) of a dodecapeptide library through whole-cell biopanning, has been reported to discover linear peptides with preferential binding to murine M2-like macrophages vs. M1-like macrophages. Further investigation for the target-binding characterization of isolated peptides indicated that strong candidates for binding remain consensus across the biological replicates (Liu et al., 2015). In addition, performing parallel biopanning experiments in biological replicates has been demonstrated to enable comparative analyses that can prove beneficial in identifying conserved target-binding motifs. This is supported by discovering confirmed binding motifs and distinguishing specific target-binding sequence clusters from non-specific background sequence clusters in duplicate NGS-based selection of a bicyclic library against human coagulation factor XIIa (Rentero Rebollo et al., 2014). These consensus motifs can provide phage display researchers with worthwhile information about the binding site of peptides toward the target. The large datasets generated by NGS provide a valuable means to identify target-binding motifs in the biopanning output (Braun et al., 2020). These motifs can be employed to design focused peptide libraries in which motif residues are kept fixed, while other positions are partially or fully randomized. Motif-based library design allows for constructing smaller and smarter libraries by concentrating on sequence features known to be enriched in a previous selection on the target (Molek et al., 2011). Thus, searching in a smaller sequence space with more relevance for target binding diminishes the likelihood of the erroneous identification of non-specific binders and increases the chance of discovering high-affinity binders or functionally relevant sequences. The NGS-based motif analysis can become more powerful by performing motif analysis not only on the biopanning output but also on the naïve library to identify background motifs. These background motifs are non-specific, and if found in the biopanning output, they should be excluded from the list of motifs for further investigation. An amplified naïve library (with no selection on the target) sequenced by NGS can also be used as a control to identify non-genuine motifs and filter them out of the candidate motifs for binding characterization.

Another interesting strategy to minimize the amplification-induced bias, which can be achieved by using NGS for the analysis of phage display selection data, is to skip the amplification of phage particles. In this approach, whole phage particles eluted from selection are directly subjected to a polymerase chain reaction (PCR). The phage capsid proteins are disassembled during the denaturation step of PCR, leading to the phage DNA being released and exposed to the PCR reaction. This approach, which is called “whole phage PCR”, has been used for the direct sequencing of phages retrieved from the selection on mycobacteria (Ngubane et al., 2013), and its efficiency has been assessed by using quantitative PCR (qPCR) (Villequey et al., 2017). This method completely omits the amplification of phage particles and allows for amplification-free selection of phage display libraries for identifying target-binding peptides. Therefore, it diminishes the amplification-associated bias that can misdirect the interpretation of biopanning data.

## 9 Concluding remarks

In recent decades, phage display has played an indispensable role in developing peptide-based diagnostics and therapeutics. There is a wealth of information demonstrating the power of this technology in the discovery of ligands with potential clinical applications. However, utilizing this biological display methodology to identify target-specific binders is plagued by some obstacles that bring about failed phage display selections. The emergence of non-specific binders isolated unwantedly during selection of the library and enriched undesirably during amplification of the selected phage pool represents the major hurdle of phage display selection, resulting in the increased ratio of misinformation (noise) to information (signal) in the biopanning output. Despite the large number of publications in the area of phage display reporting the successful identification of target-specific binders, the scientific literature suffers from a paucity of information on the challenges that researchers might face in isolating promising binders and how to surmount these obstacles. Thus, the scientific community needs to further address these challenging facets of phage display-based ligand discovery. In recent years, the rise of NGS and its integration into phage display have led to a deeper understanding of the information content of the phage population recovered from biopanning and its changes during further rounds of selection and amplification. Beyond doubt, the knowledge acquired from the large-scale sequencing of the naïve unselected, selected, and amplified phage display libraries can provide a detailed and firm grasp of the compositional characteristics of phage display libraries and how their sequence composition is altered by moving forward through rounds of biopanning. Furthermore, this knowledge can offer valuable insights into the biases associated with amplification and the approaches to minimize this bias. This awareness can finally serve to devise more efficient protocols for phage display selections and have a more rigorous evaluation of biopanning data. Here, we tried to shed light on some of the complications encountered during phage display selection and discuss them within the context of peptide libraries. We hope this article stirs further debates to illuminate the dark side of the affinity selection of phage display libraries.
